# High-capacity and selective ammonium removal from water using sodium cobalt hexacyanoferrate†

**DOI:** 10.1039/c8ra07421f

**Published:** 2018-10-08

**Authors:** Yong Jiang, Kimitaka Minami, Koji Sakurai, Akira Takahashi, Durga Parajuli, Zhongfang Lei, Zhenya Zhang, Tohru Kawamoto

**Affiliations:** Graduate School of Life and Environmental Sciences, University of Tsukuba 1-1-1, Tennodai Tsukuba Ibaraki 305-8572 Japan; Nanomaterials Research Institute, National Institute of Advanced Industrial Science and Technology (AIST) 1-1-1 Higashi Tsukuba 305-8565 Japan tohru.kawamoto@aist.go.jp

## Abstract

A new NH_4_^+^ adsorbent with high capacity and selectivity, sodium cobalt(ii) hexacyanoferrate(ii) (NaCoHCF, Na_*y*_Co(ii) [Fe^2+^(CN)_6_]_*x*_·*z*H_2_O), was prepared. The adsorption performance was investigated by varying the mixing ratio of [Fe(CN)_6_]^4−^ to Co^2+^ during synthesis, *R*_mix_. The ammonia capacity was found to be proportional to *R*_mix_, indicating that the NH_4_^+^ capacity can be increased by increasing the Na^+^-ion content in NaCoHCF. To conduct a detailed study, we prepared homogeneous nanoparticles by flow synthesis using a micromixer with *R*_mix_ = 1.00. Even on the addition of a saline solution (NaCl) with an Na^+^-ion concentration of 9350 mg L^−1^, the capacity was maintained: *q*_max_ = 4.28 mol kg^−1^. Using Markham–Benton analysis, the selectivity factor, defined by the ratio of equilibrium constants for NH_4_^+^ to that for Na^+^, was calculated to be *α* = 96.2, and 4.36 mol kg^−1^ was found to be the maximum capacity. The high selectivity of NaCoHCF results in good NH_4_^+^-adsorption performance, even from seawater. In comparison with other adsorbents under the same conditions and even for a NH_4_Cl solution, NaCoHCF showed the highest capacity. Moreover, the coexisting Na^+^ caused no interference with the adsorption of ammonium by NaCoHCF, whereas the other adsorbents adsorbed ammonia only slightly from the saline solution. We also found that the pores for NH_4_^+^ adsorption changed their sizes and shapes after adsorption.

## Abstract 2

High capacity and selectivity of NH_4_ adsorption achieved by the crystal structure optimization.

## Graphical abstract

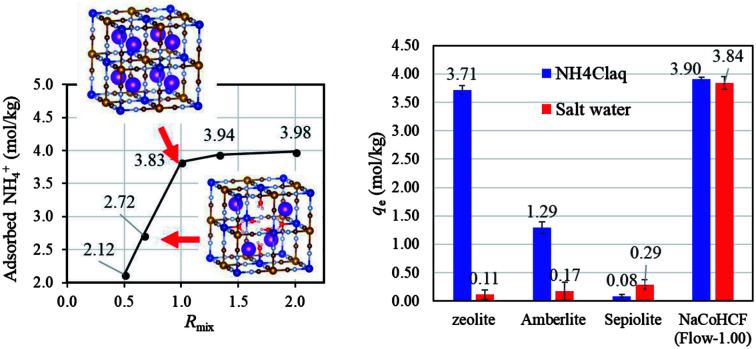

## 1. Introduction

Ammonium, NH_4_^+^, is a widely synthesized chemical, most of which is used as a fertilizer. This distributed ammonium is oxidized to nitrite, NO_2_^−^, and then further converted to nitrate, NO_3_^−^, in rivers, lakes, and oceans through nitrification processes. When humans and animals drink water containing ammonia (NO_3_^−^ ≥ 45 mg L^−1^ or NO_3_–N ≥ 10 mg L^−1^),^1^ they can become ill or even die. In aquatic systems, especially those near densely populated settlements or large-scale livestock facilities, the high concentrations of ammonia in water cause concern. Excess ammonia in aquatic systems can cause eutrophication, harmful algal blooms, and anoxic conditions in estuaries, rivers, and even the coastal environment. Consequently, biodiversity, fisheries, and overall ecosystem health are adversely affected by this N nutrient imbalance.^2–4^ Furthermore, N_2_O,^1^ a byproduct of ammonium oxidation, is an important greenhouse gas and ozone-depleting agent. The Intergovernmental Panel on Climate Change (IPCC) has estimated that the N_2_O emissions from the open ocean represent 3.8 Tg N per year, 35% of the total natural emissions.^5^

In fact, various nations have set standards for ammonia in effluent or environmental water. For example, the U.S. Environmental Protection Agency (EPA) lowered the limit in 2013 for aquatic life in ambient water bodies to 17 and 1.9 mg L^−1^ total ammonia nitrogen one-hour and 30 day averages, respectively.^6^ China and India have also established standards for effluents of 15–50 and 50 mg L^−1^ ammonium nitrogen.^7,8^ Reducing the ammonium concentration is also important for biogasification technology with anaerobic digestion because the digestion is inhibited by high concentrations of ammonium. Depending on the conditions, 1500–5000 mg L^−1^ of total ammonium nitrogen can cause the slow down or failure of digestion.^9,10^ For the removal of NH_4_^+^ from wastewater or digestion liquids, methods for the selective removal of NH_4_^+^ are necessary because various ions coexist in these solutions.

For the uptake of NH_4_^+^, removal with an adsorbent is an easily controllable and highly efficient method. Strong acid cation (SAC) exchange resins, analogs of Amberlite IR-120 (Alfa Aesar, UK), show the highest adsorption capacity, reaching 5.34 mol kg^−1^ for aqueous solution without coexisting cations and using glass-packed bed columns.^11^ Nevertheless, SAC resins have low selectivity to NH_4_^+^ when other competing ions are present. He *et al.* studied alkaline-activated and lanthanum-impregnated zeolites^12^ and found a maximum adsorption capacity, *q*_max_, of 1.54 mol kg^−1^ in a pure water solution. However, the removal efficiency, *P*_R_, decreased from 90% to 36% in the presence of Na^+^. Soetardji *et al.* reported that sodium-hydroxide-modified zeolite mordenite has *q*_max_ = 3.0 mol kg^−1^ in aqueous solution and that *P*_R_ decreased from 81% to 66.9% when competing with other ions.^13^ Guaya *et al.* studied a hydrated aluminum-oxide-modified zeolite,^14^ which showed *q*_max_ = 2.14 mol kg^−1^ in aqueous solution and *P*_R_ = 12% with coexisting Na^+^ ions.

Thus, conventional adsorbents have lower selectivity. As mentioned above, for the removal of NH_4_^+^ from wastewater or the digestion liquids, adsorbents with high-selectivity are crucial. In particular, for digestion liquids, a large capacity is also essential because the NH_4_^+^ concentration is quite high. For these reasons, the development of new adsorbents with both large capacity and high selectivity is desirable.

In our earlier study, we found that potassium copper hexacyanoferrate, KCuHCF, has a high ammonium adsorption capacity of 1.94 mol kg^−1^, as well as high selectivity for dissolved ammonia.^15^ Potassium copper hexacyanoferrate (KCuHCF), a metal hexacyanoferrate (MHCF), is a Prussian blue analog. MHCFs have the chemical composition of A_*y*_M[Fe(CN)_6_]_1−*x*_·*z*H_2_O, where A and M, respectively, denote alkali and transition metals, and *x* indicates the concentration of [Fe(CN)_6_] vacancies. With respect to MHCFs, many researchers have studied their use in catalysis,^16–18^ electrodes in secondary batteries,^19–22^ electrochromism,^23–27^ sensors,^28,29^ gas storage,^30–32^ photomagnets,^33–35^ and adsorbents for radioactive Cs^+^ ions.^36–40^ The ionic radii of hydrated Cs and NH_4_ are similar (3.29 and 3.31 Å, respectively). Therefore, assuming a size-based adsorption model, MHCFs could also have substantial adsorption capability for NH_4_^+^.^41^

**Fig. 1:**
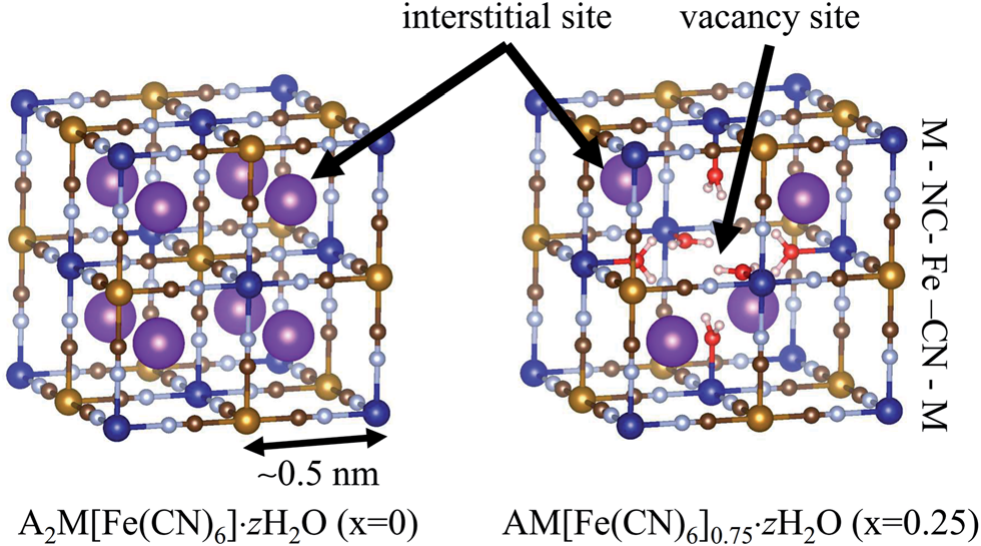
The crystal structure of metal hexacyanoferrate (MHCF, A_*y*_M[Fe(CN)_6_]_1−*x*_·*z*H_2_O) with different vacancy concentrations, *x*. Purple spheres represent A^+^ cations, the ion-exchange species in NH_4_^+^ adsorption.

The NH_4_^+^-adsorption mechanism is affected by the porous network in MHCFs. The crystal structure of MHCF is shown in Fig. 1, where two kinds of adsorption sites exist. One is an interstitial site, a cubic confined space surrounded by eight metal sites and twelve cyano-groups. Its pore size is less than 0.5 nm. The other is a vacancy site, represented as [Fe(CN)_6_] vacancies. In the case of KCuHCF, both K^+^ and NH_4_^+^ are located in the interstitial sites, as shown by Rietveld analysis of the X-ray diffraction (XRD) patterns.^15^ In addition, NH_4_^+^ is adsorbed *via* the ion-exchange with the A^+^ ions (K^+^ in the case of KCuHCF).^15^ Therefore, to enhance the adsorption capacity, [Fe(CN)_6_] vacancies should be eliminated. This is because the number of A^+^ ions increases as the number of vacancies decreases because of charge balance. The other reason is that the vacancy site does not play a role in NH_4_^+^ adsorption.

Based on these considerations, in this paper, we investigated the use of another MHCF, sodium cobalt hexacyanoferrate (NaCoHCF), to enhance the adsorption capacity. The most important difference between KCuHCF and NaCoHCF is the difference between Cu and Co. In the case of KCuHCF, KCuHCF with fewer [Fe(CN)_6_]^4−^ vacancies causes material instability in the aqueous solution.^42^ However, with substitution with Co, the introduction of a small number of [Fe(CN)_6_]^4−^ vacancies becomes possible. Additionally, the affinity of MHCF for the mono-cation is known to depend on the hydrated radius, implying that utilization of Na^+^ instead of K^+^ can increase the NH_4_^+^ adsorption performance.

Our study has two parts. The first is a composition-dependent study. Five kinds of NaCoHCF-nanoparticles (NaCoHCF-NPs), Na_*y*_Co[Fe(CN)_6_]_*x*_·zH_2_O, were synthesized by changing the molar concentration ratio of the reagent solution (*R*_mix_) using a batch method. The second part is a detailed study of *R*_mix_ = 1.00. Quantitative analysis into the adsorption capacity and selectivity was conducted. The changes to the crystal structure are also discussed. By comparison with earlier studies, we found that our NaCoHCF exhibits the a very high capacity when using the batch-adsorption method. Particularly for NH_4_^+^ adsorption from saline solutions, the benefits of NaCoHCF are enhanced by its high selectivity. We also demonstrate its potential for recyclability.

## 2. Experimental section

### 2.1 Synthesis of NaCoHCF-NPs

First, NaCoHCF-NPs with compositions of Na_4*x*−2_Co[Fe(CN)_6_]_*x*_ (water omitted) were prepared according to the following chemical reaction.Co(NO_3_)_2_ + *x*Na_4_[Fe(CN)_6_] → Na_4*x*−2_Co[Fe(CN)_6_]_*x*_ + 2NaNO_3_

To study the composition dependence, the NaCoHCF-NPs were synthesized using a batch method by mixing two aqueous solutions of Na_4_[Fe(CN)_6_]·10H_2_O (Wako Pure Chemical Ind., Ltd.) and CoCl_2_·6H_2_O (special grade from Wako Pure Chemical Ind., Ltd.) with different molar concentration ratios (*R*_mix_ = 0.50, 0.75, 1.00, 1.50, and 2.00). Here, *R*_mix_ represents the mixing ratio of the concentration of [Fe(CN)_6_]^4−^ to that of Co^2+^. The suspension was shaken using a multi shaker (SI-300C; AS One Corp.) for 3 min at 1700 rpm and room temperature. After shaking, the slurry solutions were centrifuged. The slurries were washed at least five times with Milli-Q water. They were dried under vacuum at 60 °C for 48 h.

**Scheme 1:**
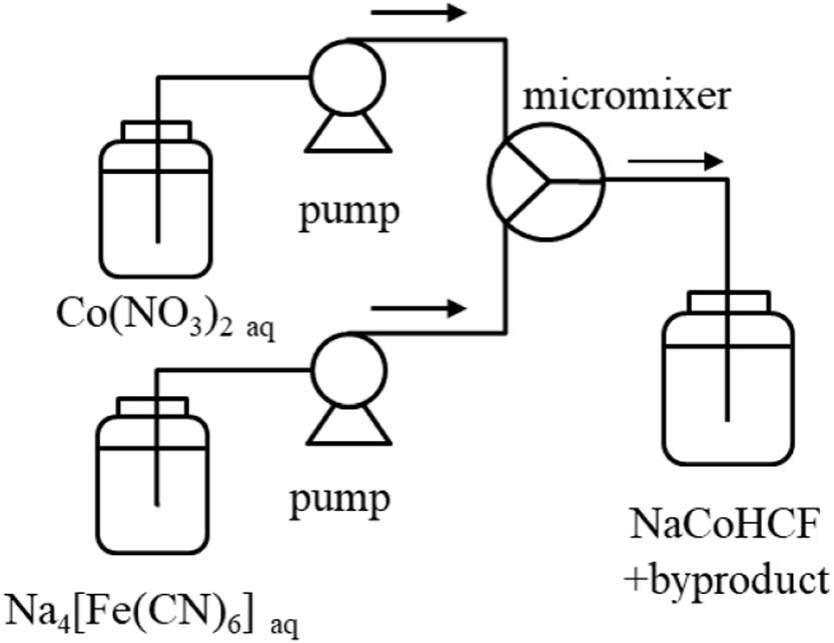
Schematic view of the synthesis of NaCoHCF by a micromixer.

For detailed studies conducted with a fixed composition, we prepared NaCoHCF-NP samples using a flow synthesis method to guarantee the homogeneity of the particle size and chemical composition.^36^ The NaCoHCF-NPs, denoted Flow-1.00, was synthesized by mixing 0.4 mol L^−1^ solutions of the Na_4_[Fe(CN)_6_]·10H_2_O and Co(NO_3_)_2_·6H_2_O (special grade from Wako Pure Chemical Ind., Ltd.) in a Y-type micro-mixer with a hole of *Φ* 250 μm, as shown schematically in Scheme 1. The mixed concentrations were the same as those for Batch-1.00. The flow rates of the two solutions were set to be equal. The total flow rate was 40 mL min^−1^. The obtained slurries were washed using a hollow fiber rinse system (DBW-24; OCT Science Co., Ltd.) to remove the NaNO_3_ byproduct. Then, the NaCoHCF-NPs were dried in vacuum at 60 °C for 72 h.

### 2.2 Characterization of NaCoHCF-NPs

The crystal structures of Flow-1.00 were studied before and after NH_4_^+^ adsorption using an X-ray diffractometer (D2 Phaser; Bruker Analytik GmbH, Germany) with Cu Kα (*λ* = 1.54 Å) radiation in the 2*θ* range of 5–60° at 30 kV and 10 mA. A Si (311) double-crystal monochromator was used to monochromatize the incident beam while reducing the high harmonics of the monochromatic beam. The XRD patterns were analyzed using the Pawley method to determine the space group and the lattice constants. For adsorption, a 500 mg L^−1^ NH_4_^+^ aqueous solution was used. Other conditions are described in Section 2.3. The crystallite sizes were estimated using Scherrer analysis of the XRD patterns, assuming a Scherrer constant of 0.94.^43^ Sample images were obtained using a field-emission scanning electron microscope (FE-SEM, S-4800; Hitachi Hitec Corp.) with 5 kV accelerating voltage after Pt–Pd coating using an ion sputter coater (E-1030; Hitachi Ltd., Japan). The chemical compositions and leaching concentration of CN^−^ into treated water were determined using a Microwave Plasma-Atomic Emission Spectrometer (MP-AES, 4100; Agilent Technologies Inc., USA) with pre-decomposition using microwaves (MW, Multiwave 3000; PerkinElmer Inc., USA). The hydration numbers in each sample were ascertained through thermogravimetric analysis (Thermo Plus EVO2; Rigaku Corp.). The specific surface areas of several samples were estimated by fitting the Brunauer, Emmett, and Teller (BET) equation to the N_2_ adsorption isotherms obtained at 77 K. The typical pre-treatment condition was 100 °C for 24 h.

### 2.3 NH_4_^+^ adsorption tests

To evaluate the composition dependence, a batch-shaking method was used to evaluate the NH_4_^+^ adsorption capacity of the NaCoHCF-NPs, as shown schematically in Fig. S1(a).† The method was conducted as follows: 40 mg of an NaCoHCF sample (Batch-0.50, 0.75, 1.00, 1.50, or 2.00) was added to a 40 mL aqueous solution of NH_4_Cl with a NH_4_^+^ concentration of 90 mg L^−1^. The suspension was shaken using a multi shaker (SI-300C; AS One Corp.) at 600 rpm for 180 min at 30 °C. After shaking, the supernatant was obtained *via* centrifuging and further separation using a 0.45 μm filter (MCE syringe filter; Membrane Solutions). The NH_4_^+^ concentration in the supernatant was evaluated using ion chromatography (IC, 883 Basic IC plus; Metrohm AG).

For a detailed study using a fixed composition, the conditions were the same except that the NH_4_^+^ concentration was changed to 1–1000 mg L^−1^ in the NH_4_Cl aqueous solution and saline solution. The saline solution was prepared with 9350 mg L^−1^ of Na^+^ ions (NaCl, special grade from Wako Pure Chemical Ind., Ltd.), where the Na^+^ concentration was set to match that of Daigo's artificial seawater SP (Wako Pure Chemical Ind., Ltd.), 9348 mg L^−1^.

For comparison, the NH_4_^+^ adsorption properties of synthetic zeolite (A-3, powder, through 75 μm, Wako Pure Chemical Industries Ltd.), sepiolite (Omi Mining Co., Ltd., Japan), and Amberlite IR-120 (H) (Alfa Aesar, UK) were also investigated. All samples were produced with no pretreatment, such as drying, before the adsorption testing. The NH_4_^+^ concentration in the aqueous NH_4_Cl and in the saline solution were both set to 500 mg L^−1^. To remove the effects of Na^+^ from the NH_4_^+^ measurements, all saline solutions were diluted and distilled to trap only NH_4_^+^ ions before measurement by IC. When the concentration of NH_4_^+^ was higher than 150 mg L^−1^ after distillation, the solutions were diluted five times for IC measurement. Other solutions were measured directly by IC.

### 2.4 Evaluation of recyclability

The potential for recyclability was also investigated. The experimental setup is shown in Fig. S1(b).† A membrane filter with NaCoHCF-NPs was prepared for the flow test. The Flow-1.00 powder was mixed with 2 mL Milli-Q (3.76 mg mL^−1^) using an ultrasonic cleaner (W-113MK-II; Honda). Then, 100 μL solutions were dropped on the membrane filter (*Φ* 25 mm, 0.45 μm pore size, JHWP01300; Merck), followed by drying at 60 °C for 2 min. Thus prepared, the solutions were set on a circular plastic plate of *Φ* 25 mm with a hole of *Φ* 5 mm in the middle. They were pasted on the film for effective adsorption–desorption. An FT-IR spectrometer (iD1 transmission iS5; Nicolet Biomedical Inc.) was used to confirm adsorption and desorption of NH_4_^+^. For the adsorption test, the NH_4_^+^ solution of 500 mg L^−1^ was flowed through the NaCoHCF-NP-dipped membrane for 30 min at the rate of 0.2 mL min^−1^. For the desorption test, a NaCl solution of 5 mol L^−1^ was similarly flowed for 2 h at a rate of 1 mL min^−1^.

## 3. Results and discussion

### 3.1 Composition dependence

The dependence of the chemical composition on the mixing ratio, *R*_mix_, is presented in Table 1. For *R*_mix_ < 1.00, *x* is an almost equal to *R*_mix_. The value of *y*, the number of Na^+^ ions in NaCoHCF, also increased. In contrast, when *R*_mix_ > 1.00, the chemical composition was almost unchanged, demonstrating that the composition can be controlled by changing the reaction *R*_mix_ to *x* < 1.

**Table 1:** The mixing ratio, *R*_mix_, in synthesis and the chemical compositions and crystal structure of the NaCoHCF-NP samples

Samples	*R*_mix_	*x*	*y*	*z*	Chemical compositions	Space group	*a* (Å)	*b* (Å)	*c* (Å)
Batch-0.50	0.50	0.57	0.49	3.64	Na_0.49_Co_1.00_[Fe(CN)_6_]_0.57_·3.64H_2_O	*P*2_1_/*m*	11.83	9.52	7.47
						*R*3̄*c*	12.77	12.77	29.06
Batch-0.67	0.67	0.63	0.74	3.41	Na_0.74_Co_1.00_[Fe(CN)_6_]_0.63_·3.41H_2_O	*P*2_1_/*m*	11.66	9.27	7.28
						*R*3̄*c*	12.97	12.97	25.77
Batch-1.00	1.00	0.87	1.65	2.84	Na_1.65_Co_1.00_[Fe(CN)_6_]_0.87_·2.84H_2_O	*R*3̄*c*	7.43	7.43	17.46
Batch-1.33	1.33	0.90	1.71	3.00	Na_1.71_Co_1.00_[Fe(CN)_6_]_0.90_·3.00H_2_O	*R*3̄*c*	7.45	7.45	17.45
Batch-2.00	2.00	0.90	1.75	3.36	Na_1.75_Co_1.00_[Fe(CN)_6_]_0.90_·3.36H_2_O	*R*3̄*c*	7.43	7.43	17.46
Flow-1.00	1.00	0.82	1.46	3.79	Na_1.46_Co_1.00_[Fe(CN)_6_]_0.82_·3.79H_2_O	*R*3̄*c*	7.39	7.39	17.55
Flow-1.00 after ads.	1.00					*Fm*3̄*m*	10.16	10.16	10.16

The crystal structure of NaCoHCF depends on the chemical composition (see Fig. S2†). When *R*_mix_ ≥ 1.00, the crystal structure is rhombohedral (*R*3̄), as reported.^44,45^ On the other hand, the structure for *R*_mix_ = 0.50 is unclear. Earlier reports described the space group as monoclinic (*P*2/*m* or *P*2_1_/*m*)^46^ or cubic (*Pm*3̄*m*).^47,48^ In our case, the XRD pattern is explained as a mixture of *R*3̄*c* and *P*2_1_/*m* structures, as shown in Table 1. The NaCoHCF with *R*_mix_ = 0.67 is also explainable as a mixture. Such a mixture could be the result of the batch synthesis because homogeneous synthesis is difficult using the batch method, resulting in the fluctuation of the chemical composition.

The BET surface areas of Batch-0.50, −0.67, and 1.00 were evaluated to be 28, 76, and 46 m^2^ g^−1^, respectively. The N_2_ isotherms are shown in Fig. S3 in the ESI.† These values are much smaller than those of other Prussian blue analogs synthesized for gas adsorption. For example, that of Co[Fe(CN)_6_]_0.60_ was 848 m^2^ g^−1^. The smaller surface area originated from the inclusion of Na^+^ cations. Because the interstitial sites of NaCoHCF are fully or partially occupied by Na^+^, it would be impossible for N_2_ to penetrate into the NaCoHCF lattice. This presumption is also supported by the fact that the averaged pore diameter is comparable to the particle size, as shown later in the analysis of Flow-1.00.

**Fig. 2:**
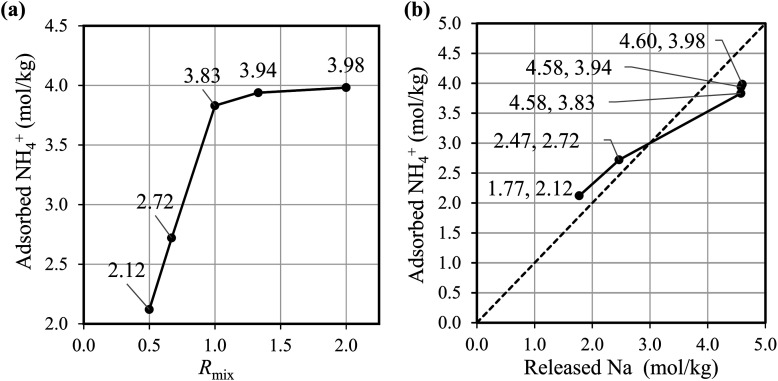
(a) Amount of adsorbed NH_4_^+^ by the batch-synthesized NaCoHCF with different compositions. (b) The relationship between adsorbed NH_4_^+^ and released Na^+^. The adsorption experiment was conducted at initial NH_4_^+^ of 90 mg L^−1^, 30 °C, and at 600 rpm for 3 h.

Fig. 2(a) shows that the NH_4_^+^ adsorption capacity improved with increasing *R*_mix_. The amount of adsorbed ammonia of Batch-2.00 is about twice that of Batch-0.5. Fig. 2(b) shows that the amount of Na^+^ from the adsorbent has an almost linear correlation with the NH_4_^+^ adsorption amount, indicating the NH_4_^+^ adsorption occurred through ion exchange with Na^+^. These results demonstrate that the increase in the Na^+^ composition in NaCoHCF enhances the NH_4_^+^ adsorption capacity, and that NaCoHCF retains its structure even after long-term shaking in water. However, for *R*_mix_ > 1.00, the adsorption capacity increased by only 2.9–3.9% from *R*_mix_ = 1.00 because the upper limit of *x* is 1.0.

### 3.2 Detailed study with flow-synthesized NaCoHCF with *R*_mix_ = 1.00

Based on results of the composition dependence of the NH_4_^+^ capacity, we chose to conduct a detailed study of NaCoHCF-NPs with *R*_mix_ = 1.00 because we obtained the desired chemical composition by using this value of *R*_mix_ and because it showed sufficiently high capacity. For our detailed study, we used the flow-synthesized sample, Flow-1.00, to avoid the fluctuation of the chemical composition and particle size. Table 1 shows that the chemical composition of Flow-1.00 is almost identical to that of Batch-1.00. The adsorption kinetics was studied at an initial NH_4_^+^ concentration of 500 mg L^−1^, 30 °C, and at 600 rpm for 8 h (see Fig. S4†). The results showed that the NH_4_^+^ adsorption was almost completed in 30 min. Such fast adsorption is similar to the case of KCuHCF.^15^

Using BET analysis, we estimated the surface area to be 53 m^2^ g^−1^, which is also comparable to that of Batch-1.00, 46 m^2^ g^−1^. As described before, this value is not very high because the interstitial sites of NaCoHCF are filled with Na^+^, preventing the penetration of N_2_ into the porous network in the crystal. The average pore size was estimated to 31 nm for Flow-1.00, consistent with the size of the crystallites, as shown later.

**Fig. 3:**
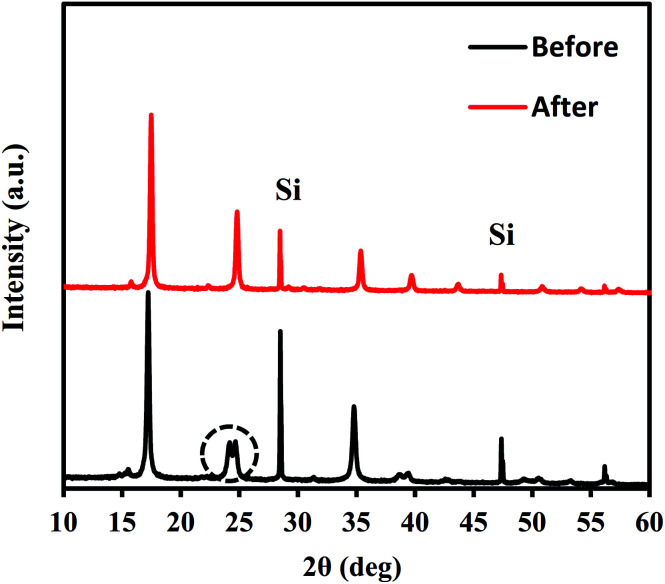
XRD patterns for Flow-1.0 with before and after adsorption with an aqueous NH_4_Cl solution containing 500 mg L^−1^ of NH_4_^+^.

After NH_4_^+^ adsorption, the crystal structure was maintained, except for a slight trigonal distortion. Fig. 3 shows the XRD patterns obtained before and after NH_4_^+^ adsorption. Some splitting of the Bragg peaks is apparent. The slight structural transformation observed is the same as that in the case of ion exchange between Na^+^ and K^+^.^45^ Before adsorption, NaCoHCF had a rhombohedral (*R*3̄*c*) structure. However, after NH_4_^+^ adsorption, it changed to a cubic lattice (*Fm*3̄*m*; *Z* = 4). Thus, the ion exchange reversibly changed the structure.

**Fig. 4:**
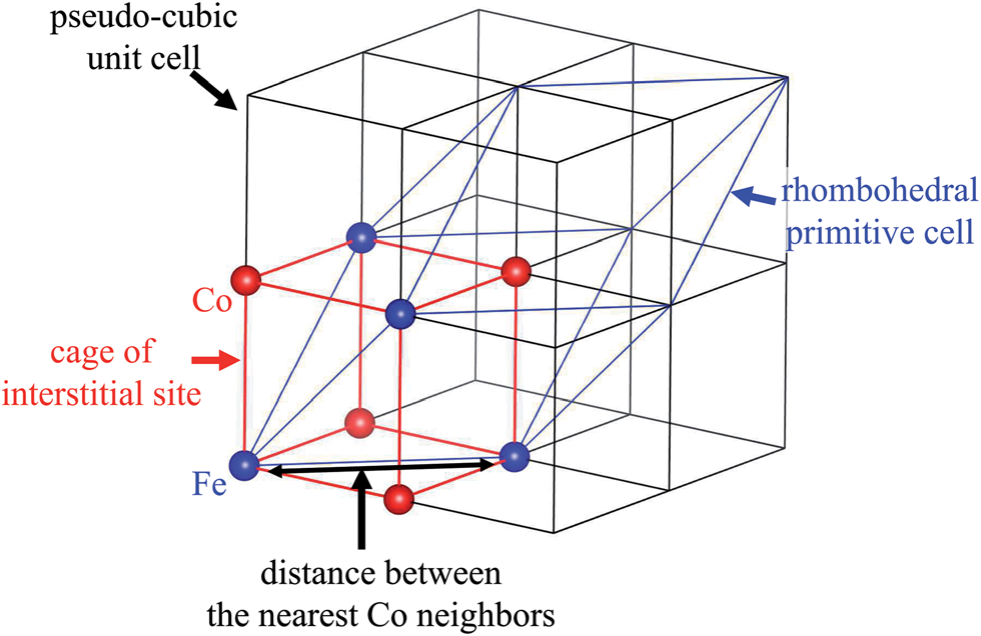
Schematic view of the relationship between the cage of the interstitial site, the cubic unit cell, and the primitive cell of the rhombohedral symmetry.

The structural change also influenced the shape and size of the interstitial sites in which Na^+^ or NH_4_^+^ are located. The lattice parameters of the crystal are shown in Table 1. A schematic view of the relationship between the interstitial site and the lattice distortion is shown in Fig. 4. Note that the crystal symmetry is different before and after the NH_4_^+^ adsorption. Before adsorption, the lattice has the rhombohedral symmetry with slight distortion from the cubic lattice. That is, it was compressed along the (1,1,1) direction. In contrast, after adsorption, the crystal maintained the cubic structure. This difference is consistent with previous reports of the ion exchange between Na^+^ and K^+^ but not NH_4_^+^.^45^

Because the symmetry of the crystal is different before and after NH_4_^+^ adsorption, we investigated the distance between the nearest Co neighbors and the volume of the (pseudo)-cubic cage of the interstitial site to evaluate the changes to the interstitial sites. Before NH_4_^+^ adsorption, the distance between the nearest Co neighbors was calculated as the length of the unit vector of the rhombohedral primitive cell, 7.24 Å. On the other hand, after NH_4_^+^ adsorption, the separation was reduced to 7.18 Å, resulting in reduction in the volume of the cage from 138 to 131 Å^3^. It is interesting that the cage became smaller although the ionic radius of NH_4_^+^ is larger than that of Na^+^. This could be due to the difference in the H_2_O accompanying the cations. Because the interaction with H_2_O is stronger with Na^+^ than that with NH_4_^+^. Therefore, before NH_4_^+^ adsorption, H_2_O is adsorbed along with Na^+^, even if cage expansion is energetically disadvantageous. This is consistent with the previous studies of the electrochemical injection of Na^+^ into copper hexacyanoferrate thin films.^49^

**Fig. 5:**
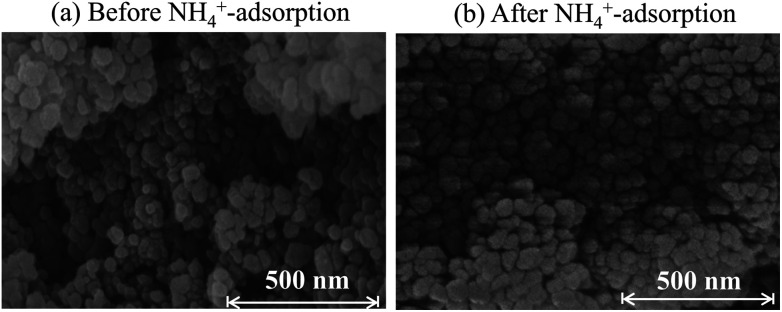
SEM images for Flow-1.00 (a) before and (b) after adsorption of 500 mg L^−1^ NH_4_^+^ in pure water solution.

No nanoparticle degradation occurred during adsorption. The crystallite sizes estimated by Scherrer analysis of the XRD patterns (Fig. 3) before and after sorption were, respectively, 37.9 and 51.7 nm. This result is consistent with the SEM images in Fig. 5. The particle sizes estimated using SEM images were 33 ± 10 and 47 ± 13 nm. The data indicate no degradation, but there is a possibility of some particle growth. The reason for the growth remains unclear, but it could be due to the immobilization of the Co^2+^ and [Fe(CN)_6_]^4−^ ions eluted from the adsorbent onto the other part of the adsorbent. If so, the adsorbent would retain the eluted species. The surface morphology in SEM images shows no marked change after adsorption. Furthermore, we also evaluated the release of CN^−^ after adsorption. The concentration of CN^−^ in solution was only 0.33 mg L^−1^, sufficiently smaller than effluent standard in Japan, 1 mg L^−1^.^50^

**Fig. 6:**
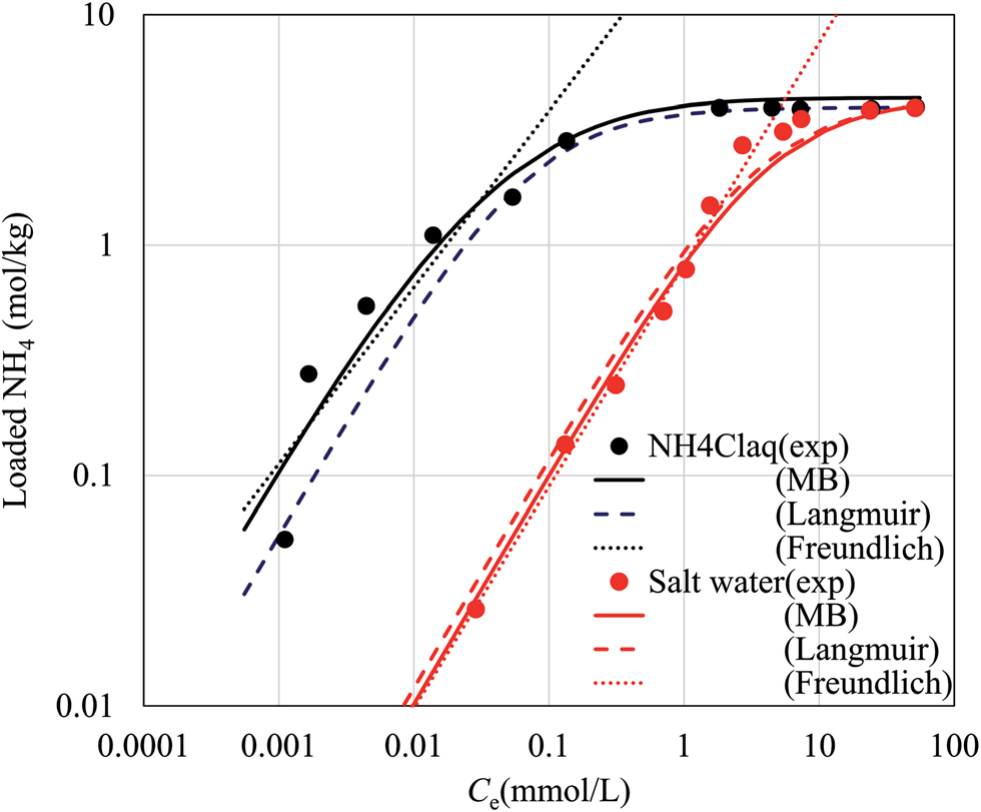
Adsorption behavior of NH_4_^+^ by the flow-synthesized NaCoHCF with *R*_mix_ = 1.00 with curves fit to the Markham–Benton (solid lines), Langmuir (broken line), and Freundlich (dotted line) equations. The experimental points are represented by closed circles. Black and red symbols represent NH_4_Cl aqueous solution and salt water, respectively.

Fig. 6 shows the NH_4_^+^ adsorption isotherms in aqueous NH_4_Cl solution and that in aqueous saline solution. In the saline solution, the concentration of the Na^+^ solution was set to 9350 mg L^−1^, the same as that of artificial seawater. The curves fit to the Langmuir, Freundlich, and Markham–Benton equations are also shown in Fig. 6. The fitting parameters for each equation are shown in Table 2. Concerning the Langmuir and Freundlich equations, individual fitting parameters were obtained for the NH_4_Cl_(aq)_ and saline solutions.

**Table 2:** The fitting parameters with the Langmuir, Freundlich, and the Markham–Benton equations for the NH_4_^+^ adsorption using the flow-synthesized NaCoHCF with *R*_mix_ = 1.00

Langmuir				Freundlich				Markham–Benton		
NH_4_Cl_(aq)_		Saline		NH_4_Cl_(aq)_		Saline		NH_4_Cl_(aq)_ & saline		
*K* (L mol^−1^)	*q*_max_ (mol kg^−1^)	*K* (L mol^−1^)	*q*_max_ (mol kg^−1^)	*K*_f_ (mol kg^−1^)	1/*n*	*K*_f_ (mol kg^−1^)	1/*n*	*q*_max_ (mol kg^−1^)	*K* (L mol^−1^)	*K*′ (L mol^−1^)
13.99	3.97	0.28	4.28	22.27	0.77	0.82	0.96	4.36	24.84	0.26

The Langmuir equation is given by1
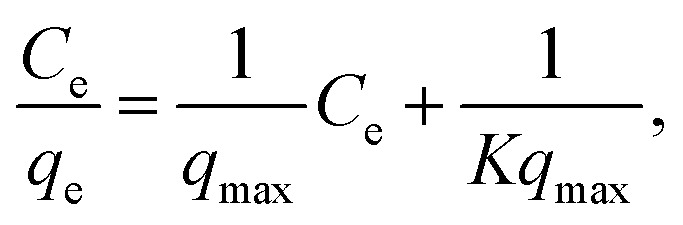
where *C*_e_, *q*_e_, *q*_max_, and *K* respectively represent the NH_4_^+^ concentration in solution at equilibrium, loaded NH_4_^+^ in the adsorbent, maximum adsorption capacity, and the equilibrium constant. The same data are also shown with other axes in Fig. S5,† and the adsorption behavior fits the Langmuir equation well. We also carried out fitting to the Freundlich equation,

2 *q*_e_ = *K*_f_C^1/*n*^.

For the Freundlich equation, only the region where the loaded NH_4_^+^ concentration was less than 1.95 mol kg^−1^, about a half the maximum capacity, was considered because the Freundlich equation is only suitable far from saturated loading. However, in this region, the Freundlich equation also well reproduced the experimental data.

In the fitting of the Langmuir or Freundlich equations, we used different parameters for NH_4_Cl_(aq)_ and the salt solutions because the effect of the coexistent Na^+^ ions can only be modeled by changing the fitting parameters. For a more quantitative evaluation of the effect of the coexistent Na^+^ ions, we also considered the Markham–Benton model for a solution with multi-alkali cations. As a type of extended Langmuir equation, the Markham–Benton equation^51^ was used to examine adsorption isotherms for multiple components to estimate the ease of desorption of the adsorbents. The results also provide some understanding of the selectivity of the sorbents for some ions. The Markham–Benton equation is3
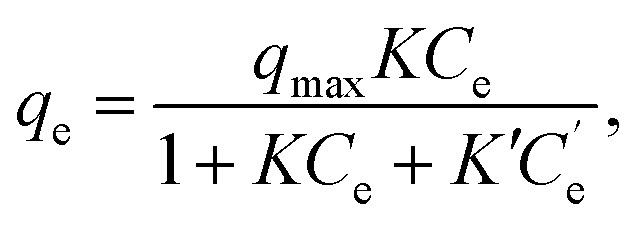
where *C*_e_, *q*_e_, and *K* respectively represent the NH_4_^+^ ion concentration in equilibrium, the adsorption capacity, and the equilibrium constant. 
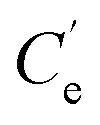
 and *K*′ respectively denote the Na^+^ ion concentration in equilibrium and the equilibrium constant.

Considering the Na^+^-ion exchange for NH_4_^+^ ions, *i.e.*, even in NH_4_Cl aqueous solution, an equal amount of Na^+^ ions would be exchanged out, adversely affecting the adsorption capacity. When we consider both sources of Na^+^ ions (those in NaCoHCF and that in the solution), the equation can be expressed by as4
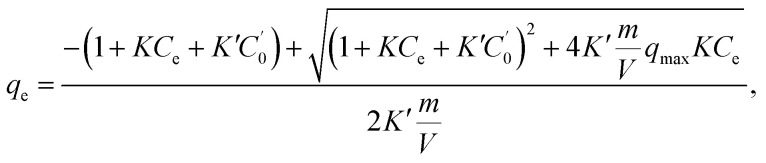
where 
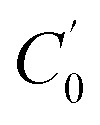
, *m*, and *V* respectively denote the initial Na^+^ ion concentration in solutions, adsorbent mass, and solution volume.

The fitting parameters, *q*_max_, *K*, and *K*′, are shown in Table 2. Fig. 6 shows that the experimental data were well fitted using the Markham–Benton model. Again, with the Markham–Benton model, we use the same parameter set for NH_4_Cl_(aq)_ and the saline solutions. A selectivity factor, *α*, defined by the ratio of equilibrium constants for NH_4_^+^ to that for Na^+^ was calculated to be *α* = 96.2, indicating the high selectivity of NH_4_^+^ against Na^+^.

Such high selectivity is expected to lead to extremely high capacity, even in an aqueous saline solution. To clarify the high capacity of NaCoHCF among the various adsorbents, we used two approaches. First, we surveyed and compared results with those of earlier studies, and we also conducted experimental investigations to assess the adsorption capacity of adsorbents in identical conditions. For the literature survey, we picked reports of adsorption tests carried out using a batch style because column-style tests generally report higher capacities, rendering a comparison of results difficult between batch-style tests and column tests.

Information from earlier studies is presented in Table 3 for the adsorption capacity of adsorbents for NH_4_^+^ from aqueous solution in batch style, indicating that the NaCoHCF (Flow-1.00) capacity exceeds that of all earlier reports. However, as described above, SAC resins have been reported to have high capacities, although these values were achieved using the column method without coexistent cations. For comparison under the same conditions, we evaluated the respective adsorption capacities of a synthetic zeolite, Amberlite IR-120(H) as a representative SAC, sepiolite, and NaCoHCF (Flow-1.00). Even in the NH_4_Cl aqueous solution (500 mg L^−1^-NH_4_), NaCoHCF exhibited the highest capacity, whereas Amberlite adsorbed only 1.29 mol kg^−1^. This result is due to the difference between column and batch tests. The capacity evaluated with a batch test sometimes larger than that with a column test, and sometimes smaller. When the contact time is short in the column test, the capacity would be underestimated. On the other hand, with bad selectivity of the adsorbent, the capacity in a batch test would be smaller than that in a column test. In the batch test, the cations A^+^ are exchanged into the solution on the NH_4_^+^ adsorption and remain in the system, resulting in the possibility of the reverse reaction (A^+^ adsorption–NH_4_^+^ desorption).

**Table 3:** NH_4_^+^ adsorption capacities of adsorbents in aqueous solutions evaluated in batch experiments reported in the literature

Adsorbent	Maximum capacity (mol kg^−1^)	Ref.
Alkaline activated and lanthanum-impregnated zeolite	1.54	12
Sodium hydroxide modified zeolite mordenite	3.0	13
Natural zeolite	2.36	14
Modified natural zeolite	2.14	14
Dowex 50w-x8	2.64	52
Sepiolite	3.70	53
Carbon nanotubes	0.95	54
Poly ligand exchanger resin	2.51	55
Cation exchange resin	0.81	56
KCuHCF	1.94	15
NaCoHCF (Flow-1.00)	4.36	This study

**Fig. 7:**
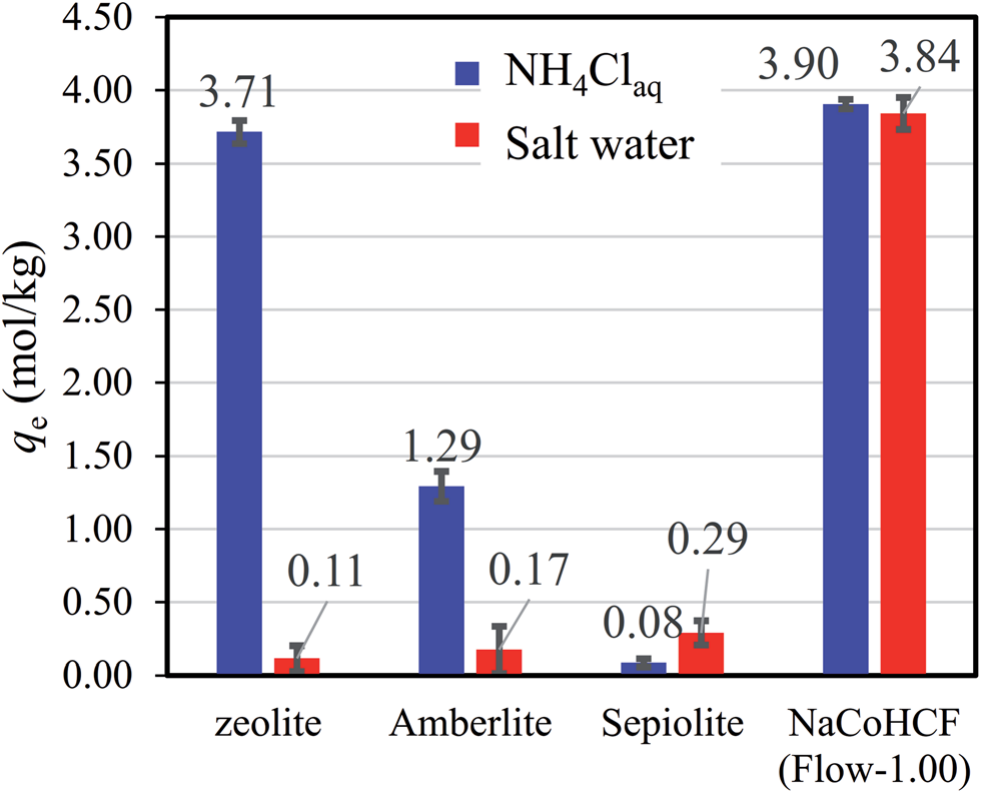
Adsorption capacity of various adsorbents in NH_4_Cl aqueous solution (500 mg L^−1^-NH_4_^+^) and saline solution with a concentration fixed at 9350 mg L^−1^-Na.

The benefits of NaCoHCF were amplified in the case of aqueous saline solution with 9350 mg L^−1^-Na and 500 mg L^−1^-NH_4_. Fig. 7 shows that NaCoHCF has an adsorption capacity that is almost identical to that of the case without Na^+^, whereas the other adsorbents showed little adsorption.

**Fig. 8:**
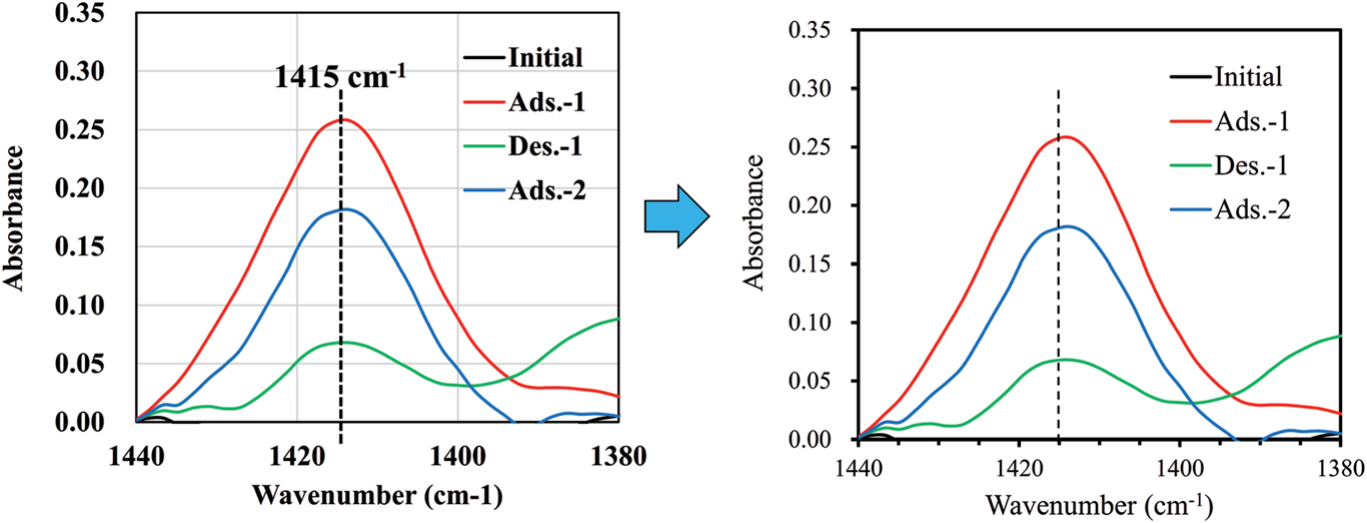
FT-IR spectra showing the changes in the peak-height of NH_4_^+^ adsorbed onto Flow-1.00 with sorption and desorption.

Although our main aim, the preparation of an NH_4_-adsorbent with high capacity and high selectivity, has been achieved, the recyclability of the adsorbent is also important for practical use. Therefore, finally, we demonstrate the potential for recyclability by attempting desorption tests. Fig. 8 shows that the adsorption–desorption–adsorption process was confirmed by measuring the infrared absorption corresponding to NH_4_-vibration mode at around 1415 cm^−1^.^15^ Using the continuous flow of NaCl solution for desorption, the peak height was found to decrease to 28% the original value before flow, indicating the potential for NaCoHCF recyclability. Next, further study on the sorption performances, including the column sorption, quantitatively reusability test would be conducted.

Finally, we mention concerning the cost of the materials. As mentioned above, NaCoHCF is synthesized only mixing two solutions immediately, resulting in the suppression of the manufacturing cost. For NH_4_ removal from salt water, NaCoHCF would be cost effective in comparison with Amberlites and zeolites, despite the utilization of the cobalt, one of the rare metals. This is because the amount of the adsorbent can be drastically decreased with its high selectivity.

## 4. Conclusions

Na_*y*_Co[Fe(CN)_6_]_*x*_·*z*H_2_O (NaCoHCF) was synthesized using a batch method and various chemical compositions. Synthesis was also carried out using a flow method and a fixed composition. The adsorption capacity increased as the number of [Fe(CN)_6_] vacancies decreased and the material was stable in water. Such stability is unlike that of copper hexacyanoferrate. We compared the NH_4_^+^ adsorption performance with other high-capacity adsorbents under the same conditions: zeolites, Amberlite ion-exchange resin, and sepiolite. The results show that NaCoHCF exhibited the highest capacity in NH_4_Cl aqueous solution. Using aqueous saline solutions with a Na^+^ ion concentration of 9350 mg L^−1^, the benefits of using NaCoHCF are enhanced drastically. The Markham–Benton model revealed the high selectivity for NH_4_^+^ against coexisting Na^+^. In addition, ammonium desorption from the adsorbent (enabling recycling) was demonstrated using an NaCl aqueous solution.

## Author contributions

The manuscript was written through contributions of all authors. All authors have given approval to the final version of the manuscript.

## Conflicts of interest

The authors declare no competing financial interest.

## Abbreviations

SAC: Strong acid cation
MHCFs: Metal hexacyanoferrates
NaCoHCF-NPs: NaCoHCF-nanoparticles

## Supplementary Material

RA-008-C8RA07421F-s001
