# Supplementary material for: High-capacity and selective ammonium removal from water using sodium cobalt hexacyanoferrate
Source: RSC Adv. 2018 Oct 8;8(60):34573–81. doi: 10.1039/c8ra07421f (PMC9086977; doi:10.1039/c8ra07421f)
Supplement: RA-008-C8RA07421F-s001 [file RA-008-C8RA07421F-s001.pdf]

Electronic Supplementary Material (ESI) for RSC Advances.
This journal is © The Royal Society of Chemistry 2018

Supporting Information

Effective ammonium removal from water using sodium cobalt hexacyanoferrates with high capacity and selectivity

Yong Jiang,[‡,†] Kimitaka Minami,[†] Koji Sakurai,[†] Akira Takahashi,[†] Durga Parajuli,[†] Zhongfang Lei,[‡] Zhenya Zhang[‡] and Tohru Kawamoto[*†]

[†]Nanomaterials Research Institute, National Institute of Advanced Industrial Science and Technology (AIST), 1-1-1 Higashi, Tsukuba, 305-8565, Japan.

[‡]Graduate School of Life and Environmental Sciences, University of Tsukuba, 1-1-1, Tennodai, Tsukuba, Ibaraki, 305-8572, Japan.

[*]Corresponding authors.

Email address: tohru.kawamoto@aist.go.jp (T. Kawamoto)

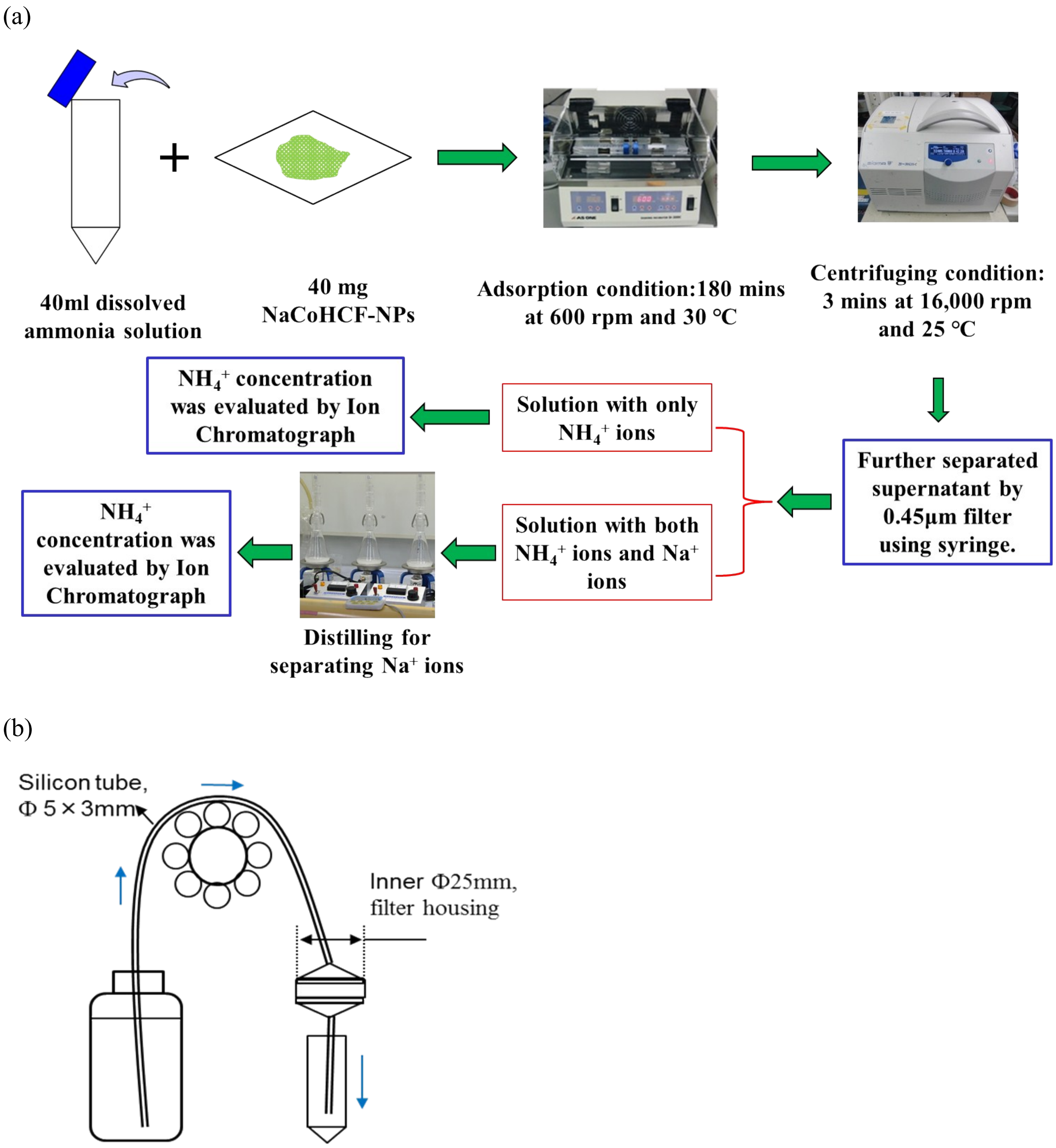

Figure S1 Schematic view of the experiment. (a) the procedure of the adsorption test. (b) Schematic view of the experimental setup for the investigation of the recyclability.

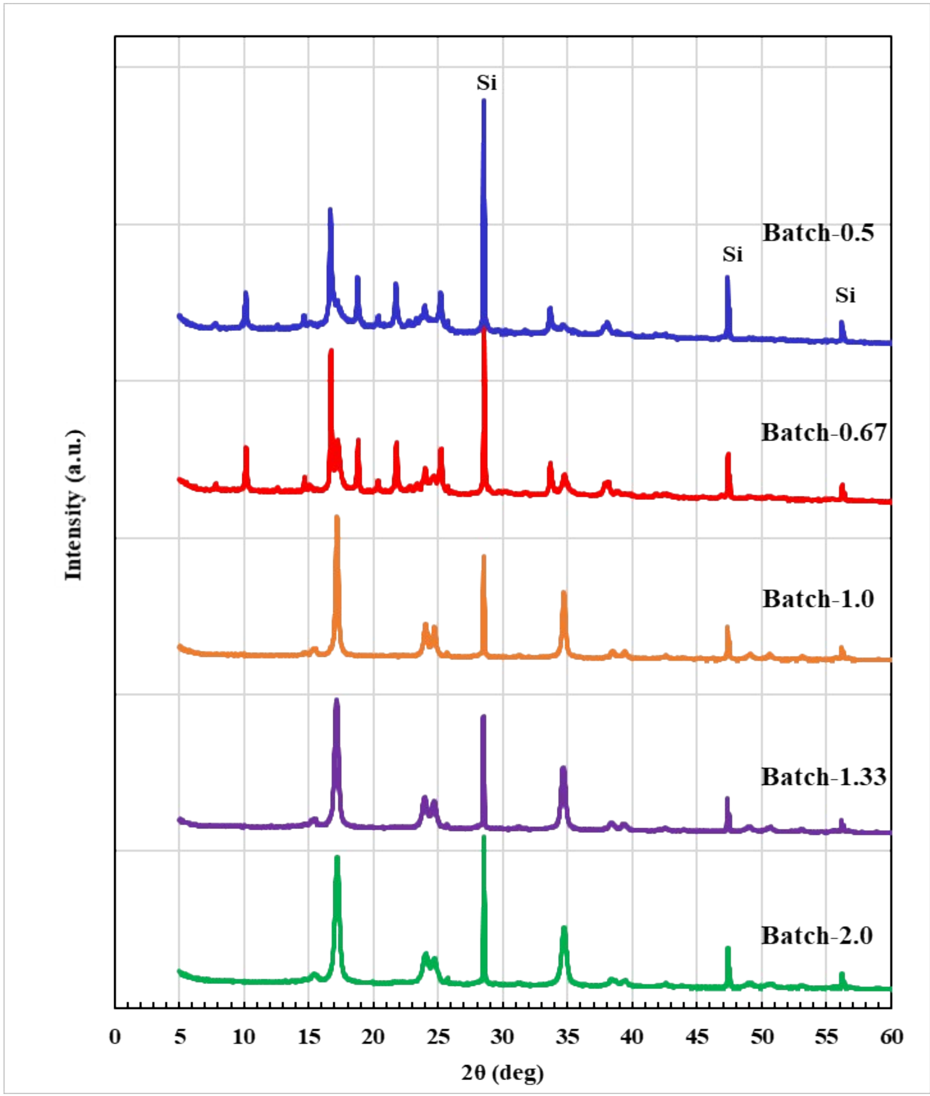

Figure S2 XRD patterns for Batch-samples. "Si" represents the peak corresponding to the Si powder mixed to the sample for the angle standard.

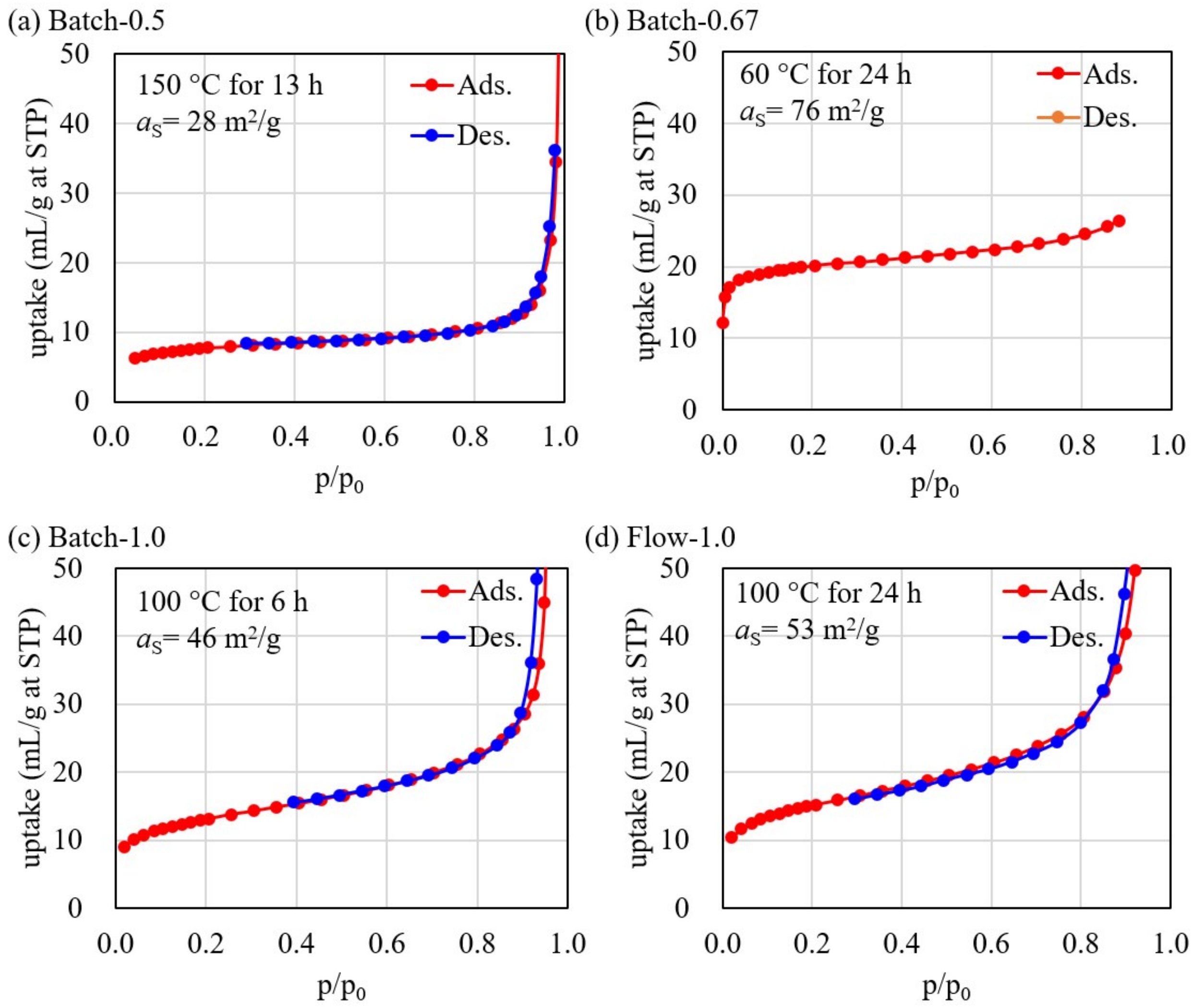

Figure S3. N_2-isotherm of NaCoHCF at 77 K

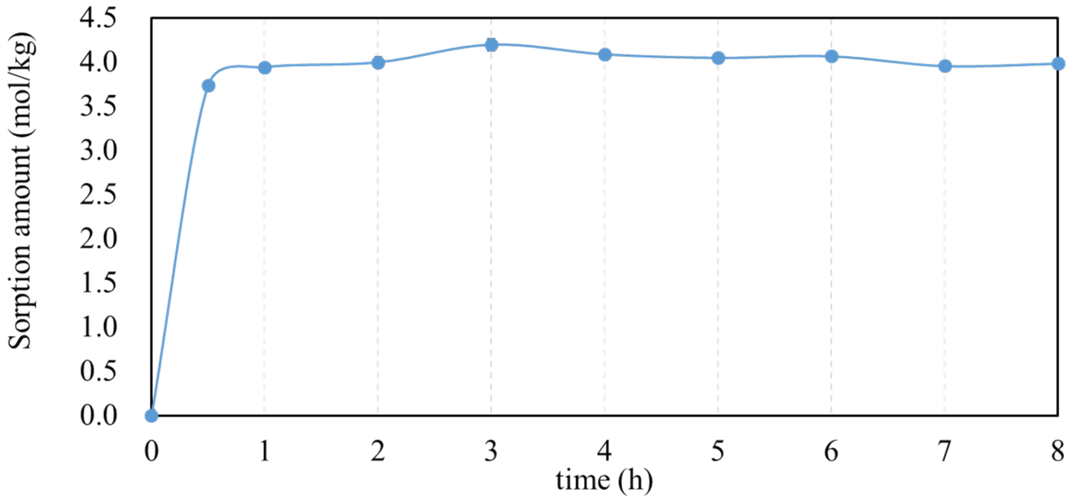

Figure S4 Adsorption kinetics of ammonia onto Flow-1.00 at 30 °C. Initial NH_4^+ = 500 mg/L, temperature = 30 °C, solid/liquid =1:1000, 600 rpm. Dilution ×500. The standard deviation is so small that it lies within the mark.

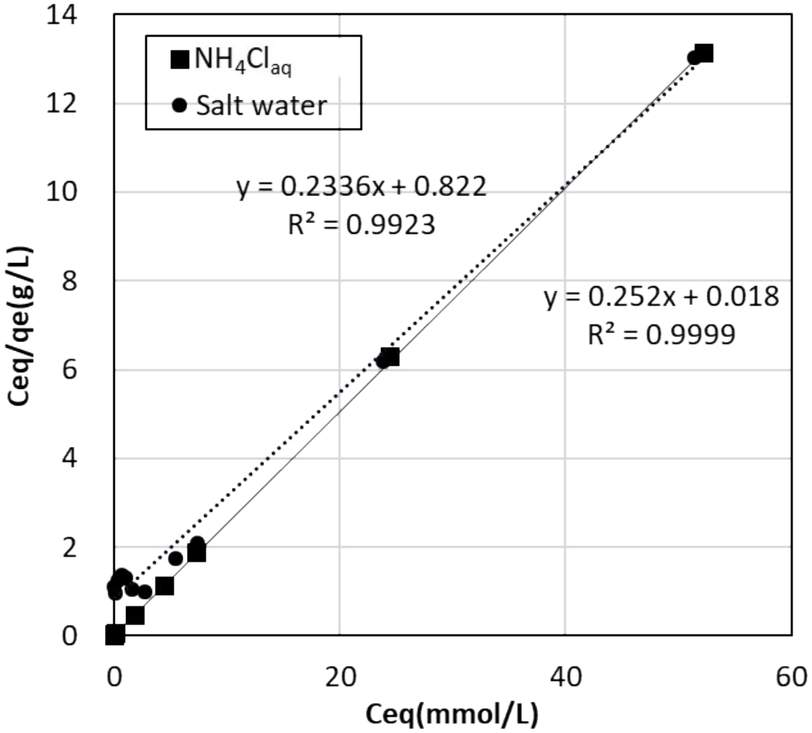

Figure S5 Adsorption behavior of NH_4^+ by flow-synthesized NaCoHCF with R_{mix}=1.00 in NH_4Cl aqueous solution and salty aqueous solution with the fitting curves by Langmuir liner model. The adsorption test was done with mixing for 3 hours with 600rpm at 30°C. The adsorbent was added into the solution at the concentration of 1-1,000 mg/L.
